# Traumatic dilaceration of permanent central incisor

**DOI:** 10.11604/pamj.2022.41.296.33533

**Published:** 2022-04-12

**Authors:** Devendra Ishwarlal Nagpal, Ayushi Shashikant Gurharikar

**Affiliations:** 1 Department of Paediatric and Preventive dentistry, VSPM's Dental College and Research Centre, Nagpur, Maharashtra, India

**Keywords:** Dilaceration, dental trauma, surgical exposure, orthodontic traction, pediatric dentistry

## Image in medicine

A 10-year-old male child reported to our department with a chief complaint of a nonerupted tooth in the upper front region of jaw. The patient's mother gave a history of trauma to anterior teeth in upper jaw 6 years back due to a road traffic accident, resulting in the intrusion of the left primary central maxillary incisor. The intruded tooth reappeared spontaneously and exfoliated normally. In the present scenario, clinical examinations showed impacted permanent maxillary left central incisor and the presence of a small hard nodule in the palatal aspect and in the labial vestibule of the same tooth. A Cone-Beam Computed Tomography (CBCT) radiographic examination was carried out in order to diagnose the cause of an impacted tooth and revealed dilaceration of permanent left central incisor along the length of a tooth. Hence, the final diagnosis was made as impacted dilacerated permanent left central incisor (due to trauma). Management of impacted dilacerated permanent teeth was done by surgical exposure with orthodontic traction.

**Figure 1:**
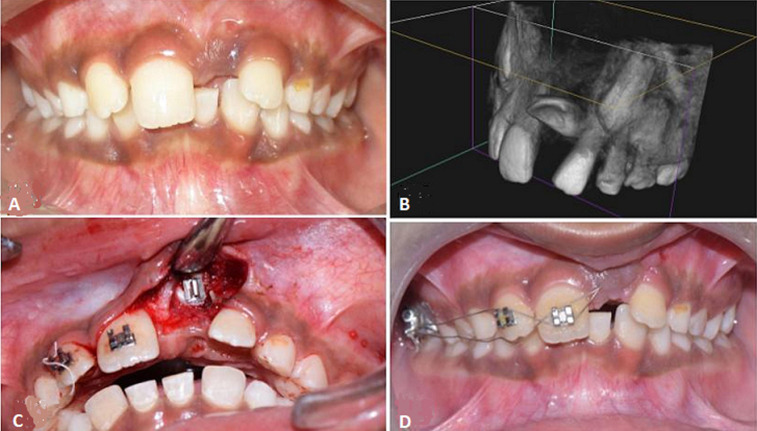
A) pre-operative clinical examination showing unerupted left permanent central incisor; B) perspective section of cone-beam computed tomography image showing an impacted tooth root located palatally with a large part close to palatal cortical bone; C) surgical exposure of impacted tooth followed placement of orthodontic brackets; D) orthodontic traction for inducing eruption of left permanent central incisor

